# Optimizing Extracellular Vesicles for Cardiac Repair Post-Myocardial Infarction: Approaches and Challenges

**DOI:** 10.3390/biom16010058

**Published:** 2025-12-30

**Authors:** Yanling Huang, Han Li, Jinjie Xiong, Xvehua Wang, Jiaxi Lv, Ni Xiong, Qianyi Liu, Lihui Yin, Zhaohui Wang, Yan Wang

**Affiliations:** 1 Department of Cardiology, Union Hospital, Tongji Medical College, Huazhong University of Science and Technology, Wuhan 430030, China; m202476238@hust.edu.cn (Y.H.);; 2 Hubei Key Laboratory of Biological Targeted Therapy, Union Hospital, Tongji Medical College, Huazhong University of Science and Technology, Wuhan 430030, China; 3 Hubei Provincial Engineering Research Center of Immunological Diagnosis and Therapy for Cardiovascular Diseases, Union Hospital, Tongji Medical College, Huazhong University of Science and Technology, Wuhan 430030, China; 4 Key Laboratory of Biological Targeted Therapy, Ministry of Education, Huazhong University of Science and Technology, Wuhan 430030, China

**Keywords:** extracellular vesicles (EVs), EVs, engineered exosomes, myocardial infarction (MI), ischemia–reperfusion (I/R) injury, cardiac repair, targeted delivery

## Abstract

Ischemic heart disease remains the leading cause of cardiovascular mortality worldwide. In myocardial infarction (MI), extracellular vesicles (EVs)—particularly small EVs (sEVs)—transport therapeutic cargo such as miR-21-5p, which suppresses apoptosis, and other proteins, lipids, and RNAs that can modulate cell death, inflammation, angiogenesis, and remodeling. This review synthesizes recent mechanistic and preclinical evidence on native and engineered EVs for post-MI repair, mapping therapeutic entry points across the MI timeline (acute injury, inflammation, and healing) and comparing EV sources (stem-cell and non-stem-cell), administration routes, and dosing strategies. We highlight engineering approaches—including surface ligands for cardiac homing, rational cargo loading to enhance potency, and biomaterial depots to prolong myocardial residence—that aim to improve tropism, durability, and efficacy. Manufacturing and analytical considerations are discussed in the context of contemporary guidance, with emphasis on identity, purity, and potency assays, as well as safety, immunogenicity, and pharmacology relevant to cardiac populations. Across small- and large-animal models, EV-based interventions have been associated with reduced infarct/scar burden, enhanced vascularization, and improved ventricular function, with representative preclinical studies reporting approximately 25–45% relative reductions in infarct size in rodent and porcine MI models, despite substantial heterogeneity in EV sources, formulations, and outcome reporting that limits cross-study comparability. We conclude that achieving clinical translation will require standardized cardiac-targeting strategies, validated good manufacturing practice (GMP)-compatible manufacturing platforms, and harmonized potency assays, alongside rigorous, head-to-head preclinical designs, to advance EV-based cardiorepair toward clinical testing.

## 1. Introduction

MI remains a major global cause of morbidity and mortality despite improvements in timely reperfusion, with incident heart failure (HF) still frequent after MI: across contemporary cohorts, post-MI HF occurs in roughly 10–15% of patients at 1 year, escalating to nearly 25% in high-risk populations [1,2,3]. Although primary percutaneous coronary intervention reduces acute mortality, there is still no proven adjunct that consistently limits ischemia–reperfusion (I/R) injury or downstream microvascular obstruction (MVO) in randomized trials; large studies targeting mitochondrial permeability-transition pore opening or using remote ischemic conditioning showed no clinical benefit [4,5,6]. MVO detected by cardiac MRI remains a robust marker of adverse remodeling and outcomes after ST-elevation MI, underscoring the need for strategies that protect both myocardium and the microvasculature [7,8]. Unlike single-target pharmacologic or conditioning approaches, EVs deliver pleiotropic cargo capable of simultaneously addressing cardiomyocyte death, inflammation, and microvascular injury—potentially overcoming the limitations of single-mechanism interventions [9,10,11,12]. In this setting, EVs can transfer cardioprotective cargo and modulate post-MI repair in vivo; preclinical work shows EVs derived from cardiac or mesenchymal sources reduce infarct size and improve function, and bioengineering can further enhance targeting and potency [9,10,11,12]. Engineering strategies span (i) optimizing donor cell sources, (ii) preconditioning donor cells, (iii) molecular and surface engineering of EVs, and (iv) carrier engineering to enhance EVs delivery; collectively, these approaches have improved EV biodistribution and functional recovery in rodent MI models, and EV-based therapies have also shown benefit in selected large-animal (porcine) MI studies, but translation has not yet progressed to human MI trials and therefore remains at a preclinical stage [10,11,12,13,14]. Collectively, converging evidence supports EVs—especially engineered EVs—as a modular, microenvironment-responsive platform to address both myocyte death and microvascular injury after MI, but future trials must resolve manufacturing, dosing, and targeting at clinical scale [12,13,14].

## 2. Basic Mechanisms: EVs Biology and MI Pathophysiology

EVs are membrane-bound nanovesicles (~30–150 nm) that carry proteins, lipids, and nucleic acids within a protective lipid bilayer, enabling transfer of this cargo between cells and contributing to intercellular signaling in cardiovascular disease [15,16]. In MI, necrotic cardiomyocytes release damage-associated molecular patterns such as HMGB1, which activate pattern-recognition receptors and trigger an innate immune cascade characterized by rapid neutrophil influx within hours, followed by recruitment and phenotypic reprogramming of monocytes–macrophages over several days; these innate immune cells critically shape infarct expansion, resolution of inflammation, and scar formation [17,18,19]. EVs can modulate several steps in this injury–inflammation–repair sequence through distinct mechanisms, including anti-apoptotic, pro-angiogenic, and immunomodulatory effects, as demonstrated across preclinical models.

Anti-apoptotic effects of EVs have been primarily linked to microRNA cargo that inhibits cell death pathways. For instance, in rodent ischemia–reperfusion models, mesenchymal stromal/stem cell (MSC)–derived EVs enriched in miR-21a-5p exhibit anti-apoptotic effects by targeting pro-apoptotic genes; when miR-21a-5p is deleted or inhibited in the EVs source, cardioprotection is markedly attenuated, whereas restoring miR-21a-5p expression or delivering miR-21a-5p–loaded EVs rescues these benefits in vivo [20].

Building on these protective mechanisms, the pro-angiogenic effects of EVs support vascular repair and tissue perfusion post-MI. MSC–derived EVs also promote angiogenesis through miR-21a-5p [20], while reparative M2 macrophage–derived EVs enhance angiogenesis and improve functional recovery, in part via transfer of miR-132-3p that targets the anti-angiogenic protein thrombospondin-1 (THBS1); in contrast, pro-inflammatory macrophage EVs carry microRNAs and proteins that tend to sustain inflammatory signaling and impair vascular repair [21,22]. Similarly, the endothelial microRNA miR-126—strongly pro-angiogenic—contributes to cardioprotection when delivered via EVs: exosomes loaded with miR-126 (alone or in combination with miR-146a) enhance neovascularization, reduce infarct size, and improve cardiac function in rat MI and ischemia–reperfusion models, and plasma exosomes enriched in miR-126a-3p after remote ischemic preconditioning confer protection against MI/R injury [23,24]. These findings are corroborated in large-animal models, where EVs from cardiosphere-derived cells (CDCs) delivered into infarcted pig hearts reduce scar size and improve left ventricular function, supporting a causal role for CDC-EVs in promoting repair [10].

Immunomodulatory effects of EVs further influence the inflammatory milieu, with macrophage-derived EVs playing a pivotal role based on polarization state. Reparative M2 macrophage EVs not only drive angiogenesis but also modulate anti-inflammatory pathways, such as through Y RNA fragments that enhance IL-10 expression and secretion to confer cardioprotection [21], whereas pro-inflammatory M1 macrophage EVs perpetuate inflammation [22].

**Figure 1:**
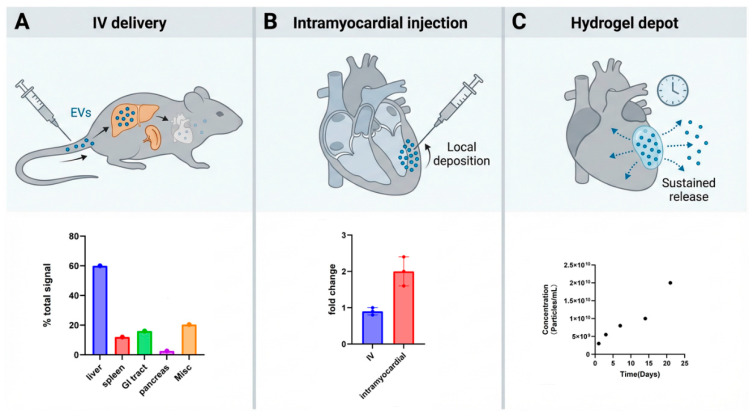
Route-dependent biodistribution/retention of EVs. (**A**) Following intravenous (IV) delivery, EVs enter systemic circulation and are predominantly sequestered by reticuloendothelial organs; the representative ex vivo organ distribution (**bottom**) illustrates liver- and spleen-dominant uptake with comparatively low cardiac signal, consistent with route-dependent EV biodistribution patterns reported in vivo. (**B**) With intramyocardial injection, EVs are deposited directly into the myocardium (**top**), resulting in higher early cardiac retention; the bottom bar plot summarizes a representative quantification showing increased heart-associated signal after intramyocardial administration compared with IV delivery (fold change relative to PBS control). (**C**) Hydrogel depot delivery places EVs within a local biomaterial reservoir adjacent to/within the myocardium (**top**), enabling sustained, localized availability over time (**bottom**), a strategy widely used to prolong EV exposure at target sites. EVs are released from the gel over time to permit cellular uptake. Cumulative EV release profile from the shear-thinning gel (*n* = 2) shows steady particle release over 21 days (**bottom**) [29].

Although these mechanisms are encouraging, natural EVs carry only modest payloads, and unmodified EVs exhibit limited intrinsic cardiac targeting. After intravenous administration in small-animal models, most EVs accumulate in the liver, spleen, and other reticuloendothelial organs, with >70% hepatic uptake and <5% cardiac accumulation in rodent biodistribution studies [25], with relatively low uptake in the injured myocardium. EVs biodistribution is shaped by EVs surface molecules and target-cell receptors: for example, exosomal integrins can direct organotropic uptake, and cell-surface heparan sulfate proteoglycans (HSPGs) act as key receptors for EV internalization in multiple tissues [26,27]. However, available data indicate that, without additional engineering, systemically delivered EVs do not selectively home to cardiac tissue [15,16]. Therefore, delivery strategy is critical: in MI models, direct intramyocardial injection or local biomaterial depots—such as injectable shear-thinning hydrogels or fibrin-based cardiac patches—substantially increase local EVs retention, reduce fibrosis, and enhance functional benefit, while encapsulation of EVs in hydrogels (including EPC- or hiPSC-derived EVs) enables sustained release in the infarct zone [28]. Furthermore, engineering EVs membranes with targeting ligands or exploiting integrin–receptor interactions has been shown to enhance uptake by desired organs, providing a rationale for developing cardiac-targeted EV formulations [1,17,27]. Altogether, these mechanistic insights support the advancement of targeted EV therapies in MI while underscoring key translational challenges in biodistribution, dosing, and delivery. “Figure 1 illustrates the route-dependent biodistribution and retention of extracellular vesicles (EVs), highlighting the differences between systemic, intramyocardial, and hydrogel-encapsulated delivery strategies. This figure visually reinforces the importance of selecting the appropriate delivery method to achieve optimal EV retention and functional benefit in myocardial infarction (MI) models.”

## 3. Therapeutic Effects of EVs in Cardiac Repair

Across rodent and large-animal models of MI, therapeutic EVs from mesenchymal, progenitor, endothelial, and immune cells produce quantifiable improvements in infarct size, left-ventricular (LV) function, and angiogenesis. In a porcine permanent-ligation MI model, seven days of intravenous human mesenchymal stromal cell (MSC)–derived small EVs reduced infarct size by approximately 30–40% at both 7 and 28 days compared with vehicle and lessened infarct transmurality and wall thinning, with relative preservation of regional LV wall thickening on cardiac MRI [30]. In acute and chronic porcine MI, intramyocardial delivery of cardiosphere-derived cell (CDC) exosomes reduced the infarct size–to–area-at-risk ratio from about 80% to roughly 60% at 48 h and attenuated the 1-month decline in LV ejection fraction (LVEF) by several percentage points compared with controls, while decreasing scar mass and increasing vessel density in treated segments [10].

In a rat MI model, human bone-marrow MSC–derived EVs significantly reduced fibrotic scar area and improved LV systolic function, accompanied by higher capillary and arteriolar densities in the peri-infarct zone on CD31/von Willebrand factor and α-smooth muscle actin staining, indicating robust pro-angiogenic remodeling [31]. Genetically engineered EVs further enhance these quantitative effects: in rats, MSC-EVs overexpressing hypoxia-inducible factor-1α (HIF-1α) produced greater improvements in LVEF and fractional shortening, smaller fibrotic area, and increased capillary counts per high-power field than both PBS and unmodified MSC-EVs, in parallel with up-regulation of VEGF and related pro-angiogenic factors [32]. Likewise, macrophage migration inhibitory factor (MIF)–induced up-regulation of miR-133a-3p in MSC-derived exosomes augmented their efficacy in rat acute MI, yielding additional gains in echocardiographic LV function, further reductions in TTC-defined infarct size, and higher CD31+ microvessel density compared with native EVs [33].

EVs from non-mesenchymal sources show similar quantitative trends. In a murine MI model, exosomes from human induced pluripotent stem cell–derived endothelial cells (hiPSC-ECs) improved myocardial contractile function, limited adverse LV remodeling, and reduced infarct size, while hiPSC-EC exosomes also directly enhanced endothelial tube formation in vitro, consistent with a pro-angiogenic profile [34]. M2 macrophage–derived exosomes injected at the onset of MI likewise improved echocardiographic indices of systolic function, reduced infarct size, and enhanced CD31+ vessel density in ischemic myocardium by delivering pro-angiogenic miR-132-3p to endothelial cells and suppressing the anti-angiogenic target THBS1 [22]. Collectively, these preclinical data indicate that natural EV preparations from MSCs, CDCs, hiPSC-ECs, and M2 macrophages can reduce infarct burden by up to roughly one-third in porcine MI and consistently improve LV function and histological measures of angiogenesis, whereas engineered EVs carrying HIF-1α or miR-133a-3p provide additional, quantifiable increments in functional recovery [1,2,3,4,5,6,7].

## 4. Engineering Strategies for Enhanced EV Efficacy

To unlock the therapeutic potential of EVs in MI, engineering strategies are being used to address key limitations—restricted effective cargo capacity suboptimal cardiac tropism,, and rapid clearance. Current efforts concentrate on (i) optimizing donor-cell sources; (ii) preconditioning donor cells to increase yield and enrich reparative cargo; and (iii) molecular engineering of EVs for enhanced efficacy to strengthen cardioprotective signaling and myocardial targeting. These strategies have led to significant improvements in the therapeutic efficacy of engineered exosomes. The table below summarizes various optimization approaches, detailing the exosome sources, engineering techniques, administration routes, experimental models, and the main therapeutic endpoints for MI treatment. It highlights the diverse strategies employed to enhance the functionality and targeted delivery of exosomes, demonstrating their potential to improve cardiac regeneration and repair. Table 1. Optimization strategies for exosome-based therapies in MI, detailing exosome sources, optimization strategies, administration routes, experimental models, and main therapeutic outcomes.

### 4.1. Optimizing Donor-Cell Sources

The donor cell determines EVs yield and baseline cargo composition, thereby shaping therapeutic potential.

Among cell sources, MSCs are the most extensively used for therapeutic EV production because they expand readily and support scalable upstream/downstream bioprocessing—e.g., serum/xeno-free microcarrier bioreactors and tangential-flow filtration (TFF)—while generally exhibiting favorable immune profiles that ease allogeneic use [27,28,34]. For the mechanism, bone-marrow MSC–derived EVs (BM-MSC-EVs) can deliver miR-21-5p that suppresses PTEN and activates PI3K/AKT in cardiac recipient cells, limiting apoptosis (shown in vitro with BM-MSC-EVs) and, when miR-21-5p–rich MSC-EVs are applied in vivo, associating with smaller infarcts and improved left-ventricular function in rodent MI models (the in vivo benefit is not BM-specific and reported with alternative miR-21-5p targets as well) [44,48]. Adipose-derived MSC-EVs (AD-MSC-EVs) support neovascularization and post-MI repair; these effects are markedly enhanced when miR-126 is engineered/enriched in the vesicles, whereas native AD-MSC-EVs cargo varies across studies and consistent endogenous miR-126 or uniformly high VEGF content is not established [11,59]. Cardiac-progenitor–cell EVs (CPC-EVs) carry cardioprotective miRNA signatures and improve early function in rat MI, and a GMP-oriented CPC-EVs workflow (including TFF and release testing) has been described, although CPC expansion/yields are typically more demanding than for MSCs [60]. Endothelial/EPC-EVs are enriched for endothelial miRNAs such as miR-126 and show pro-angiogenic activity in vivo—including improved perfusion and function after MI with sustained intramyocardial delivery—yet large-scale culture and GMP standardization remain shared challenges across EV therapeutics [61]. In practice, MSC-EVs often provide a pragmatic platform balancing scalability, immune compatibility, and reparative cargo, while CPC-EVs and EC/EPC-EVs are pursued to emphasize targeted endpoints such as cardiomyocyte survival or angiogenesis [41,42,49,52].

### 4.2. Preconditioning Donor Cells

Beyond intrinsic cell-type features, preconditioning can further bias EVs payloads toward pro-repair signals. The current preconditioning approaches mainly include: (i) hypoxic preconditioning; (ii) drug/molecular preconditioning; and (iii) genetic engineering of donor cells.

#### 4.2.1. Hypoxic Preconditioning

Culturing donor MSCs under physiological hypoxia (~1–5% O_2_ for 24–48 h) stabilizes HIF-1α expression, activates canonical hypoxia-inducible programs including upregulation of VEGF-A and CXCL12/SDF-1, enhances levels of hypoxia-responsive miR-210, and alters the molecular cargo of secreted EVs [12,13,29,35,36,62]. In rodent MI models, EVs derived from hypoxia-preconditioned MSCs reduce cardiomyocyte apoptosis and enhance post-MI cardiac function compared to those from normoxic MSCs, at least in part because hypoxia increases the loading of miR-210 and miR-125b-5p into these vesicles; knockdown of miR-125b-5p in these EVs diminishes cardioprotective effects in vivo [12,13]. For AD-MSC-EVs, hypoxia preconditioning augments angiogenic potential in vitro and in vivo, associated with increased miR-126-5p in non-cardiac diabetic wound models [29]. Collectively, hypoxia preconditioning shifts EV contents toward pro-angiogenic and pro-survival pathways (e.g., miR-210 and miR-125b-5p, with context-dependent miR-126-5p involvement), supporting improved tissue repair in preclinical MI models, though benefits vary by study and model.

#### 4.2.2. Drug/Molecular Preconditioning

Pharmacologic (drug/molecular) priming of donor cells reliably reprograms the cargo of native EVs and can augment myocardial repair in rodent acute MI models, although the magnitude and mechanisms vary by agent and context. In rats, exosomes from atorvastatin-pretreated MSCs reduce infarct size and improve function via upregulated lncRNA H19/miR-675 signaling [38], and in an independent MI study, atorvastatin-primed MSC-EVs promote anti-inflammatory macrophage M2 polarization by delivering miR-139-3p that targets STAT1, thereby enhancing post-MI healing [15]. Beyond statins, nicorandil pretreatment produces MSC-exosomes enriched in miR-125a-5p that repress TRAF6/IRF5, driving M2 polarization and improving ventricular remodeling after MI in rats [16]. Traditional Chinese medicine (TCM) pharmacology has also been applied: Tongxinluo-pretreated MSCs yield exosomes carrying higher miR-146a-5p, which targets IRAK1/NF-κB p65 to lessen cardiomyocyte apoptosis and inflammation and to improve left-ventricular function in MI [17]. Finally, nicotinamide mononucleotide (NMN) preconditioning of human umbilical cord MSCs generates EVs enriched in miR-210-3p that targets EFNA3, promoting angiogenesis and reducing fibrosis with functional recovery in rat MI [18]. Collectively, across these original MI studies, pharmacologic priming can shift native EV miRNA/lncRNA payloads toward pro-repair programs.

#### 4.2.3. Genetic Engineering of Donor Cells

Genetic modification of donor cells can be leveraged to engineer EVs toward three major therapeutic objectives in MI: suppressing cardiomyocyte death, improving cardiac targeting, and prolonging EV persistence via immune evasion. At the anti-apoptotic level, MSCs overexpressing miR-21-5p secrete EVs enriched in miR-21, which target and suppress pro-apoptotic genes such as PTEN and PDCD4 [46]. In rodent MI models, delivering these miR-21-rich EVs inhibits cardiomyocyte apoptosis and improves post-MI cardiac function, largely via EV-carried miR-21 downregulating YAP1 in recipient cardiomyocytes [63]. Similarly, EVs derived from MSCs engineered to overexpress miR-214-3p promote endothelial migration and tube formation and enhance cardiomyocyte survival under ischemia. In a rat acute MI model, treatment with miR-214-3p-enriched EVs reduces myocardial apoptosis, decreases infarct size, boosts peri-infarct angiogenesis, and improves cardiac function by transferring miR-214-3p that inhibits PTEN and activates AKT signaling [47]. Additional anti-death modifications include forced expression of the transcription factor GATA-4 in bone-marrow MSCs: exosomes from GATA-4-overexpressing cells carry increased levels of miR-330-3p, which inhibits ferroptosis in hypoxia/reoxygenation-injured cardiomyocytes by targeting the BAP1/SLC7A11 pathway [50]. Consistently, administering GATA4-engineered exosomes after MI reduces cardiomyocyte apoptosis and improves cardiac function in infarcted hearts [51]. To improve targeting to the injured heart, donor cells can be engineered to present cardiac-homing ligands on EVs membranes. For example, an ischemic myocardium-homing peptide fused to the exosomal membrane protein Lamp2b in MSCs produces exosomes that accumulate more in the ischemic heart and provide greater therapeutic benefit in MI models [54]. Likewise, donor cells expressing a cardiomyocyte-specific binding peptide fused to Lamp2b generate EVs that are taken up more efficiently by hypoxic cardiomyocytes and show enhanced cardiac retention in vivo, leading to reduced cardiomyocyte apoptosis [12]. Native homing receptors can also be exploited: cardiac progenitor cells overexpressing the chemokine receptor CXCR4 produce exosomes displaying CXCR4 on their surface; after systemic administration in MI rats, these CXCR4-bearing exosomes exhibit improved targeting to ischemic myocardium, resulting in smaller infarcts and better recovery of left ventricular function compared with control EVs [49]. Finally, engineering EVs to display “don’t-eat-me” signals can enhance immune evasion and prolong circulation. Overexpression of CD47 in EV-producing cells increases EVs surface CD47, enabling EVs to avoid macrophage-mediated phagocytosis [57]. Such CD47-decorated EVs persist longer in the bloodstream and accumulate more in the injured heart, translating into superior functional recovery after MI relative to unmodified EVs [57]. Collectively, these examples show that rational genetic engineering of donor cells to deliver anti-apoptotic cargo, cardiac-targeting ligands, or immune-evasive markers can markedly increase EV retention in infarcted myocardium and amplify their therapeutic efficacy in MI models.

### 4.3. Molecular and Surface Engineering of EVs

Beyond donor-cell programming, direct molecular engineering during or after EVs biogenesis enables precise loading of therapeutics and surface functionalization with targeting ligands. For small-molecule loading, heart-targeted EVs bearing a cardiac-targeting peptide (CTP) and co-loaded with curcumin and miR-144-3p displayed higher myocardial residence, lowered oxidative stress and apoptosis, and improved post-MI function in rodents, pointing to gains conferred by engineered surfaces plus exogenous payloads rather than native cargo alone [21]. A related two-step platform that briefly reduces mononuclear-phagocyte uptake using DOPE-PEG–CTP before administering curcumin-loaded EVs further enhanced antioxidant effects and left-ventricular performance in mouse MI, again emphasizing the value of pairing cardiac targeting with small-molecule encapsulation [39]. For protein cargo, umbilical-cord MSC–derived engineered nanovesicles covalently modified with a cardiac-homing peptide and loaded with placental growth factor (PLGF) increased cardiac retention, stimulated angiogenesis and cardiomyocyte proliferation, reduced fibrosis, and improved ventricular function in MI rodents, illustrating a feasible protein-loading path when combined with targeting chemistry [53]. Independent surface-functionalization strategies likewise improved delivery without altering native cargo: DOPE-NHS conjugation of the ischemic-myocardium homing peptide CSTSMLKAC to EVs increased cardiac accumulation and was associated with better ejection fraction, angiogenesis, and scar reduction after MI/I-R in rats [64], while exosomes presenting an ischemic myocardium-targeting peptide via donor-cell engineering achieved enhanced homing and therapeutic benefit in MI [54]. Donor-cell display of a cardiomyocyte-binding peptide (Lamp2b-fusion) increased uptake by hypoxia-injured cardiomyocytes, reduced apoptosis, and improved in vivo cardiac retention after intramyocardial delivery in rodents [4]. Collectively, these studies support molecular engineering—via exogenous small-molecule/protein loading and cardiac-focused surface functionalization—as a generalizable route to elevate EVs residence at the infarct and amplify therapeutic impact in MI.

## 5. Carrier Engineering to Enhance EV Delivery

Therapeutic performance of EVs–based interventions is often constrained by rapid systemic clearance and limited deposition at target sites. To address these barriers, this section focuses on carrier engineering to stabilize EVs, localize payloads, and enable programmable targeting while preserving biocompatibility. Current approaches fall into two categories: biomaterial carriers for localized EV delivery and synthetic or hybrid EV carrier systems.

### 5.1. Biomaterial Carriers for Localized EV Delivery

Embedding EVs in biocompatible hydrogels or scaffolds can create local depots that prolong residence and sustain release at the injury site, thereby enhancing functional benefit relative to bolus EVs. In rodent cardiac models, an injectable hyaluronic acid (HA) “ExoGel” loaded with MSC-derived exosomes and delivered into the pericardial space preserved wall thickness and reduced dilation versus controls; the same approach demonstrated feasibility and acute safety of intrapericardial delivery in a porcine model, but did not assess efficacy outcomes, aligning with the broader limitation that large-animal efficacy data remain sparse [56]. In osteoarticular repair, intra-articular EVs delivery improves cartilage structure and pain in rodent models, and combining EVs with HA can further support defect repair in rabbits, consistent with the depot concept [65,66]. Across tissues, hydrogels and related scaffolds generally act by stabilizing/retaining native vesicle cargo near the lesion rather than “increasing dose,” but head-to-head large-animal comparisons and standardized long-term safety remain sparse.

### 5.2. Synthetic and Hybrid EV Carrier Systems

Engineering carriers can add control and targeting beyond passive depots. Hybrid vesicles formed by fusing EV membranes with liposomes preserve EVs’ biocompatibility while improving nucleic-acid loading and delivery; for example, MSC exosome–liposome hybrids transfected large Cas9-GFP plasmids into human HEK293T cells more efficiently than plasmid alone, supporting the principle though in vivo efficacy remains to be established [67]. From a translational perspective, such hybrids could in principle exploit existing liposomal manufacturing platforms, but scalable, GMP-compliant fusion processes that preserve EVs surface markers and cargo composition have not yet been standardized, and batch-to-batch variability remains a concern. Clinically, their most realistic near-term application may be ex vivo gene editing or local administration where high nucleic-acid payloads are needed, rather than repeated systemic dosing of a complex chimeric biological–synthetic drug product. External-field guidance is another approach: magnetically steerable, muscle-derived exosomes tethered to ferromagnetic nanotubes were directed to dystrophic muscle in mice and enhanced tissue repair in a Duchenne muscular dystrophy model, illustrating on-demand localization after systemic dosing [68]. However, translation will require scalable production of ferromagnetic nanotubes with tightly controlled magnetic properties and rigorous toxicology of long-term nanotube retention in muscle and off-target organs. In patients, clinically acceptable magnetic-steering paradigms would likely be limited to anatomically accessible muscle groups or organs that can be bracketed by external magnets, and integration with imaging for real-time field placement will be needed. Compared with untargeted EVs infusion, magnetic guidance may be particularly advantageous when focal delivery to relatively superficial tissues is sufficient to achieve therapeutic benefit. Biomimetic cloaking further extends this concept: platelet-membrane–exosome hybrids showed superior ischemic-heart targeting with reduced macrophage clearance and improved repair in mouse MI [69]; more recently, neutrophil-derived apoptotic body–membrane–fused exosomes leveraged inflammation-homing signals to adhere to injured endothelium and improved function and remodeling in MI mice [55]. For both strategies, manufacturing at scale will depend on reliable sourcing and processing of human platelets or neutrophil-derived apoptotic bodies, stringent pathogen screening, and control of prothrombotic or proinflammatory components. In terms of clinical positioning, platelet cloaking may be preferable when prolonged circulation and homing to thrombus-associated or ischemic vascular lesions are desired, whereas neutrophil-mimetic exosomes may be better suited for acute, highly inflamed myocardial tissue where transient adhesion to activated endothelium is critical. Direct head-to-head comparisons and standardized safety assays for thrombosis, immunogenicity, and off-target inflammation will be essential before either biomimetic cloak can Sbe advanced toward early-phase clinical trials.

## 6. Manufacturing and Quality Control of Clinical-Grade EVs

Scalable, reproducible manufacturing and rigorous quality control (QC) are prerequisites for translating EV therapies to patients. Recent primary studies demonstrate that switching from planar (2D) culture and ultracentrifugation to bioreactors and modern downstream processing can increase EVs output while maintaining identity and bioactivity, whereas field guidelines (e.g., MISEV2018/2023) outline minimal characterization requirements for clinical development [70,71,72]. To place these considerations in the context of practical bioprocess design, Table 2 summarizes commonly used EVs isolation and purification methods, highlighting their typical applicability, purity, yield, and potential impact on EV biological function.

### 6.1. Scalable Production Methods

Multiple 3D culture platforms now support clinically relevant EV yields. In umbilical-cord MSCs (UC-MSCs), a hollow-fiber bioreactor increased exosome yield ~7.5-fold versus matched 2D flasks while preserving vesicle size/markers and enhancing function in vivo, providing sufficient material to support weekly intra-articular injections over a 4-week osteochondral defect study in 15 rabbits without depleting production capacity [81]. Stirred-tank reactors (STRs) with microcarriers have also been operated under xenogeneic-free conditions to enable continuous EV harvests; in one controlled STR workflow using Wharton’s jelly MSCs, the process achieved ~1.26 × 10^4^ particles per cell per day with high particle-to-protein ratios and intact uptake/activity readouts [71]. In the same system, a representative 3-day production phase from ~6 × 10^7^ cells yielded ~2.1 × 10^12^ particles, illustrating that even small-scale STR runs can supply 10^12^-level EV quantities suitable for preclinical and early-phase clinical investigations [71]. Beyond upstream culture, coupling 3D production with tangential-flow filtration (TFF) downstream has delivered order-of-magnitude gains in recovered EVs and improved bioactivity compared with 2D plus ultracentrifugation [70]. Collectively, these primary data support hollow-fiber and microcarrier-STR platforms as viable routes to mitigate the yield bottleneck relative to flasks while maintaining EV attributes within tested contexts.

### 6.2. Advanced Purification Technologies

Downstream choices involve trade-offs between throughput, purity, and scalability. Differential ultracentrifugation (DUC) is widely used but can co-isolate protein/lipoprotein contaminants from biofluids and media [75,82]. In head-to-head studies, size-exclusion chromatography (SEC)—often preceded by TFF/ultrafiltration for volume reduction—improves purity and/or recovery relative to DUC and precipitation kits, and can be completed within hours at lab scale [75,82,83,84,85]. Hybrid trains such as TFF → SEC or SEC → ultracentrifugation further reduce soluble proteins and apolipoproteins in plasma-derived sEVs [83,84]. Immunoaffinity capture (e.g., anti-tetraspanin) isolates marker-defined subpopulations but, by design, excludes other EV subsets and currently poses scale/cost constraints despite promising high-throughput formats [47,49]. Emerging label-free microfluidic/acoustofluidic approaches can separate EVs from lipoproteins continuously, but typical μL–mL per hour throughputs remain a limitation for large-volume manufacturing [86]. In practice, platform selection should align with intended clinical dose and batch size; scalable combinations such as TFF pre-concentration with SEC polishing are increasingly favored when both yield and purity are required [41,44,46].

### 6.3. Robust Quality Control (QC) Frameworks

For clinical-grade EVs, QC spans identity, purity, safety, and (ideally) potency. MISEV2018/2023 recommends orthogonal characterization of size/concentration (e.g., nanoparticle tracking analysis), morphology (TEM/cryo-EM), and protein markers (≥1 transmembrane/GPI-anchored such as CD9/CD63/CD81, plus negative markers to flag contaminants) [72,73]. Safety testing typically includes sterility, mycoplasma, and endotoxin assays under GMP-compliant conditions; recent large-scale manufacturing studies of UC-MSC sEVs demonstrate that such release panels can be met in practice, with Wharton’s jelly MSC–derived sEV batches fulfilling predefined endotoxin, mycoplasma, and sterility criteria, although the precise numeric limits remain process- and jurisdiction-dependent [71,87]. Related hollow-fiber bioreactor workflows for bone marrow MSC-EVs produced under current GMP procedures further exemplify scalable, clinically oriented manufacturing strategies [88]. Potency assays (e.g., endothelial tube formation, migration, macrophage modulation) are widely used to benchmark bioactivity during process development, and recent primary work demonstrates assayable changes in angiogenesis-related activity with defined manufacturing variables such as culture substrate stiffness or other controlled mechanical cues [89]. Nevertheless, no individual in vitro potency assay has been prospectively validated as a surrogate for clinical benefit in EVs trials, and available data linking assay readouts to patient outcomes remain sparse and indication-specific [90]. This lack of clinically validated potency metrics constitutes a major translational bottleneck, complicating cross-study comparability, dose selection, and regulatory acceptance of EVs products [90]. Together, adherence to MISEV-guided identity/purity characterization, GMP-aligned sterility, mycoplasma, and endotoxin controls, and fit-for-purpose, mechanism-informed potency assays currently provide a defensible minimum QC framework for clinical-grade EVs, but this framework will need to be iteratively refined as outcome-linked potency criteria and pharmacodynamic biomarkers are established in larger, well-controlled clinical studies [71,72,73,87,88,89,90].

## 7. Challenges and Future Prospects

Despite encouraging preclinical signals and tangible progress in EV engineering and manufacturing, several barriers still hinder the translation of EVs therapies for post-MI repair.

### 7.1. Challenges of Quality Control

EVs preparations are intrinsically heterogeneous in size, composition, and biogenesis and are frequently admixed with non-vesicular extracellular particles (e.g., lipoproteins), so heterogeneity and co-isolates themselves constitute a central QC challenge by complicating standardization, release testing, and lot-to-lot comparability [72]. From a QC standpoint, side-by-side single-particle studies show that the isolation workflows summarized in Section 6 (e.g., ultracentrifugation, SEC, density gradients, TFF-SEC, TIM4-affinity) yield EVs populations with different co-isolate burdens and detectable subpopulations and can bias single-particle readouts—for example, higher apparent “EVs-positive” flow-cytometric events after ultracentrifugation than after SEC or TIM4-based capture due to non-EVscontaminants [91]. Single-EVs analyses further demonstrate biological heterogeneity (e.g., uneven tetraspanin distribution; only a subset of vesicles carrying detectable DNA cargo), but they also reveal pronounced method-dependent detection, underscoring how difficult it is to define QC thresholds that are robust across platforms and protocols [92,93]. MISEV2023 explicitly acknowledges these issues and cautions that no single quantitative readout—including the often-used particle-to-protein ratio—can be treated as a universal purity metric; instead, it recommends context-specific multi-assay panels together with improved removal and quantitative measurement of co-isolates such as albumin and ApoB-containing lipoproteins [72,84]. Collectively, ongoing adoption of single-particle methods and quantitative immunoassays alongside advanced omics is needed to define critical quality attributes with higher precision [72,84,91], but the field still lacks consensus, operational CQAs and acceptance criteria that can be applied consistently across manufacturing processes, indications, and clinical centers, as also emphasized by recent EV therapeutics reviews that frame EVs as biopharmaceuticals whose “process defines the product” and highlight heterogeneity- and co-isolate–driven challenges for regulatory quality assessment [94,95].

### 7.2. In Vivo Targeting and Biodistribution

After intravenous dosing, native EVs display predominant liver/spleen (mononuclear phagocyte system) uptake with relatively low myocardial accumulation, as shown by quantitative imaging and ex vivo analyses in rodents; systematic reviews reach similar conclusions across models [16,64]. Surface engineering can improve—but not abolish—this pattern: cardiotropic peptide display (e.g., CSTSMLKAC fused to LAMP2b) increases cardiac localization and modestly improves post-MI function in mice, yet off-target hepatic and splenic deposition remains substantial [64,96]. Whole-body, time-resolved quantification is increasingly feasible: PET/SPECT studies (e.g., ^89Zr-labeled engineered EVs) enable route-dependent kinetic profiling in rodents and non-human primates, while iron-oxide approaches enable MRI tracking with variable sensitivity and standardization [40,54]. Together, these data support a realistic expectation that targeting augments rather than transforms biodistribution, and that quantitative nuclear imaging will be valuable for dose/route optimization prior to clinical translation [16,64,96,97].

### 7.3. Immunogenicity and Safety Assessment

Although EVs are endogenous in origin, immune effects depend on source, dose, purity, and engineering. Tumor-derived EVs can induce neutrophil extracellular traps and accelerate thrombosis in vivo, demonstrating immunostimulatory potential in certain contexts [98], and recent reviews further highlight EVs as central mediators of cancer-associated thrombosis [99]. However, long-term immunogenicity and tumorigenicity datasets for therapeutic EVs, especially engineered products, remain limited and warrant cautious interpretation of current safety profiles. Overall, rigorous control of non-native carryover (e.g., serum-derived vesicles, lipoproteins, endotoxin) and fit-for-purpose immunogenicity panels aligned with MISEV2023 are needed to de-risk development [72,84].

### 7.4. Clinical Bottlenecks

Progress to late-phase trials is constrained by cGMP manufacturing, validated analytics, and agreed potency criteria. Comparative studies show that upstream culture and isolation choices (e.g., 3D versus 2D expansion, TFF-SEC versus ultracentrifugation) shift apparent yields, purity, and composition, potentially confounding biological readouts [72,91,100]. 3D and perfusion-like systems can increase EV output and, in some reports, augment functional activity in rodent MI, but results are not uniform across platforms and indications [100]. Potency assays remain under qualification: for MSC-EVs, CD73 ecto-5′-nucleotidase activity is mechanistically attractive yet has not consistently correlated with immunomodulatory potency across lots and assays, underscoring the need for validated, fit-for-purpose functional tests rather than single surrogates [101]. Clinically, most EVs trials remain early-phase with heterogeneous endpoints and few qualified translational biomarkers, supporting calls for harmonized CQAs, process controls, and consensus endpoints (e.g., via MISEV2023 and regulator-informed guidance) [102]. Phase I EVs trials should prioritize safety and biodistribution assessments (for instance, using radiolabeled EVs to enable whole-body tracking of EVs distribution and clearance) [103]. Phase II efficacy trials should incorporate rigorous imaging endpoints, such as cardiac MRI to quantify infarct size reduction and improvements in left ventricular ejection fraction (LVEF) at ~3–6 months [104]. Additionally, consensus potency assays should be established through collaborative working groups (e.g., ISEV and ISCT) to define standardized, mechanism-informed potency metrics and accelerate regulatory acceptance of EV therapeutics.

### 7.5. Clinical Translation: Registered Interventional Studies in Cardiovascular Disease

As of December 2025, EsV-based cardiac repair remains predominantly preclinical, with only a limited number of registered interventional studies directly testing EV-containing products in cardiovascular indications (Table 3). Most are early-phase and emphasize safety/tolerability, with exploratory imaging or biomarker readouts rather than definitive clinical efficacy endpoints. For example, SECRET-HF evaluates repeated intravenous infusions of an EV-enriched secretome from iPSC-derived cardiovascular progenitor cells in patients with heart failure with reduced ejection fraction, using serious adverse events as the primary outcome over a short follow-up window, alongside exploratory bioactivity/potency and immunologic measures [105]. In the ischemic heart disease setting, a first-in-human intracoronary infusion study administers an EV-containing biological drug (PEP) immediately after PCI/stent placement, with dose-limiting toxicities and maximum tolerated dose as the primary endpoint and cardiac MRI-based scar size and ejection fraction as secondary measures [106]. Another small randomized study in CABG candidates with recent Q-wave MI and severely reduced LVEF evaluates intracoronary/intramyocardial delivery of mesenchymal stem cell–derived exosomes (with or without mitochondria), using ejection fraction and allergic reactions as primary outcomes at 3 months [107]. Beyond the heart, EV-based approaches have also been tested in vascular-complication contexts such as chronic ulcers—conditions that often overlap with peripheral ischemia—where topical plasma-derived exosomes were evaluated with ulcer size metrics as primary outcomes [108]. Collectively, these studies underscore both the promise and the current limitations of translation: the clinical pipeline is still sparse, heterogeneous in product definition and route, and largely focused on establishing safety, dosing feasibility, and measurable target engagement. Standardized product characterization aligned with MISEV principles, harmonized potency assays, and clinically meaningful endpoint strategies will be essential for EV therapeutics to progress beyond proof-of-concept. As shown in Table 3, the clinical trials involving exosome-based therapies in cardiovascular and vascular complications are still in early phases, with a focus on safety, tolerability, and exploratory endpoints.

## 8. Conclusions and Future Perspectives

The recent advancements in EVs engineering, particularly for MI therapy, show considerable promise but also underscore the need for clear priorities in translation. Studies have highlighted the potential of EVs to modulate the critical stages of post-MI repair, including inflammation, tissue regeneration, and microvascular recovery. These vesicles, derived from both stem-cell and non-stem-cell sources, have demonstrated the ability to reduce infarct size, enhance vascularization, and improve cardiac function in preclinical models. The therapeutic efficacy of EVs can be further enhanced through bioengineering techniques such as optimizing donor cell sources, preconditioning, surface ligand modification, and the use of biomaterial depots to prolong myocardial residence. For basic and translational researchers, the key takeaway is to delineate the mechanistic pathways and standardize engineering strategies so that different EVs platforms and models can be compared in a rigorous and reproducible manner.

Positioned within the broader landscape of post-MI cardioreparative strategies, EV-based therapeutics occupy a distinct middle ground between cell therapies and conventional biologics/small molecules. Compared with cell transplantation, EVs offer a cell-free, potentially off-the-shelf modality that can recapitulate key paracrine benefits while avoiding issues such as poor engraftment and cell-related arrhythmogenic risk. In contrast to single-target proteins or small molecules, EVs can deliver modular, multi-component cargo (RNAs, proteins, lipids) that may better match the multi-phase biology of post-MI repair. However, EVs share—and in some respects amplify—translational hurdles, including manufacturing and batch consistency at scale, uncertainty in effective dosing and biodistribution, and regulatory classification that depends on source, manipulation, and intended use. Clarifying these comparative advantages and constraints is essential to define where EVs are most likely to add value clinically—most plausibly as adjuncts to reperfusion and guideline-directed therapy, initially in well-defined patient subsets and with imaging-anchored endpoints.

For clinicians and trial designers, the main message is that EVs are currently best positioned as adjuncts to contemporary MI care, and that future clinical studies should refine patient selection, dosing regimens, and clinically meaningful endpoints such as infarct size and ventricular function. For bioengineers, industry partners, and regulators, the critical next steps are to converge on scalable manufacturing, robust potency assays, and pragmatic safety monitoring frameworks that can support progression to late-phase trials.

Despite these opportunities, translating EVs-based therapies into routine clinical practice remains a significant challenge. Key issues include variability in EVs preparations arising from the inherent heterogeneity of EVs populations and differences in isolation and engineering methods, as well as incomplete standardization of quality-control measures such as identity, purity, and potency assays. Looking ahead, several concrete research avenues must be pursued to address these gaps: refining EVs isolation and characterization to improve reproducibility and reduce batch-to-batch variation; advancing targeted delivery systems and dosing strategies to ensure efficient homing to ischemic myocardium; and integrating quantitative imaging approaches (e.g., PET/SPECT) to track EVs biodistribution and guide optimization of delivery routes and doses. Ultimately, the successful translation of EVs-based therapies for post-MI cardiac repair will depend on rigorous preclinical studies with well-defined endpoints, coupled with the establishment of scalable, GMP-compatible manufacturing and safety-monitoring frameworks that can support large-scale, late-phase clinical trials.

## Figures and Tables

**Table 1:** Optimization strategies for exosome-based therapies in MI.

Exosome Source	Optimization Strategy	Administration Route	Experimental Model	Main Therapeutic Outcomes	Ref.
EPC-EVs	shear-thinning hydrogel–based local delivery; sustained release/retention	IM (local myocardial injection)	Rat MI	enhanced angiogenesis; improved cardiac function; increased EV retention	[29]
BMMSC-EVs	source-cell genetic engineering (FNDC5 overexpression); anti-inflammation/macrophage polarization	IM (infarcted heart injection)	Mouse MI	attenuated inflammation/apoptosis; promoted M2 polarization; improved post-MI cardiac function	[35]
MSC-EVs	exogenous cargo loading (miR-132) via electroporation	IM (ischemic heart/peri-infarct delivery)	Mouse MI	enhanced neovascularization; preserved cardiac function; pro-angiogenic effect	[36]
ADSC-EVs	exogenous cargo loading (miR-126 + miR-146a mimics); hydrogel encapsulation (alginate derivative) for sustained release	local myocardial delivery (hydrogel-based)	Rat MI	reduced infarct size; reduced fibrosis; enhanced angiogenesis	[24]
iCM-EVs	delivery matrix optimization (Matrigel/PBS carrier) to improve retention of injected exosomes	IM (peri-infarct, multi-site injection)	Mouse MI	improved cardiac function/viability; reduced cardiomyocyte apoptosis; improved myocardial repair	[37]
Plasma-EVs	donor preconditioning (RIPC-induced exosomes); therapeutic miRNA transfer (miR-24)	direct myocardial injection	Rat I/R injury	reduced apoptosis; reduced infarct size; cardioprotection	[38]
ADSC-EVs	source-cell genetic engineering (miR-126 overexpression); pro-angiogenic enhancement	IV (tail vein)	Rat AMI	decreased inflammation/fibrosis; increased angiogenesis; reduced myocardial injury	[39]
MSC-EVs	hypoxia preconditioning; cargo enrichment via donor-cell engineering (miR-210 OE, mechanistic validation)	IM (peri-infarct/border zone)	rat MI (LAD ligation)	reduced infarct size; improved cardiac function; attenuated apoptosis	[40]
MSC-EVs	hypoxia preconditioning; surface conjugation with IMT cardiac-targeting peptide (enhanced targeting/retention)	IV (tail vein)	mouse MI (coronary ligation)	improved cardiac function; reduced cardiomyocyte death; enhanced myocardial targeting	[41]
MSC-EVs	donor-cell drug pretreatment (atorvastatin); lncRNA H19 upregulation/enrichment	IM (border zone)	rat AMI (LAD ligation)	improved cardiac function; reduced infarct size/apoptosis; enhanced angiogenesis; reduced inflammation	[42]
BM-MSC-EVs	donor-cell drug pretreatment (atorvastatin); cargo modulation (miR-139-3p mimic/inhibitor via transfection)	IM (border zone, multi-point)	rat AMI (LAD ligation)	improved LVEF/LVFS; promoted M2 macrophage polarization; enhanced cardiac repair	[43]
MSC-EVs	donor-cell pretreatment (Tongxinluo); miR-146a-5p–associated cardioprotection	IM (border zone, multi-point)	rat AMI (LAD ligation)	improved cardiac function; reduced infarct size; anti-apoptotic/anti-inflammatory; enhanced repair	[44]
hUCMSC-EVs	donor-cell pretreatment (NMN); EV optimization with miR-210-3p enrichment/functional dependence	IM (peri-infarct/border zone)	rat MI	improved cardiac function; reduced LV remodelling/fibrosis; enhanced angiogenesis; reduced apoptosis	[45]
HEK293T-EVs	genetic engineering for miR-21 enrichment; localized delivery strategy	local IM (infarct area)	rat MI	restored cardiac function; reduced injury/remodelling; pro-survival/pro-repair effects	[46]
HuMSC-EVs	donor-cell genetic engineering (miR-214 OE) for therapeutic cargo enrichment	IM (border zone)	rat MI	improved cardiac function; reduced infarct size; enhanced repair	[47]
MSC-EVs	drug preconditioning (nicorandil); pro-repair cargo shift (e.g., miR-125a-5p upregulation)	IM (border zone)	Rat AMI (coronary ligation)	improved LVEF/LVFS; reduced infarct size; reduced fibrosis/inflammation; enhanced angiogenesis	[48]
CPC-EVs	donor-cell genetic engineering (CXCR4 overexpression) to improve homing/efficacy	IV (systemic)	Rat myocardial I/R	reduced infarct size; improved cardiac function; enhanced cardiac retention	[49]
BMSC-EVs	genetic engineering (GATA-4 overexpression)	IV (tail vein)	Mouse myocardial I/R	reduced infarct area; improved LVEF/LVFS; attenuated ferroptosis	[50]
BMSC-EVs	genetic engineering (GATA-4 overexpression)	IM (post-MI injection)	Mouse MI	improved cardiac function; reduced apoptosis; enhanced angiogenesis	[51]
HEK293-EVs	surface targeting peptide (CTP) via LAMP2b display; cargo loading (curcumin); miRNA loading (miR-144-3p); co-delivery	IV	Mouse MI	enhanced heart accumulation; reduced apoptosis; improved therapeutic efficacy; improved cardiac function	[52]
UCMSC-EVs	cargo loading (PLGF); surface conjugation (CHP, covalent); engineered nanovesicles	IV (systemic)	Mouse MI	improved cardiomyocyte survival; improved cardiac repair/function	[53]
MSC-EVs	surface display of ischemic myocardium-targeting peptide (CSTSMLKAC; IMTP)	IV (tail vein)	Mouse MI	enhanced myocardial retention; improved EF/FS recovery; attenuated remodelling	[54]
MSC-EVs	biomimetic membrane fusion (neutrophil apoptotic body membrane; NAM)	IV (tail vein)	Mouse MI	reduced fibrosis; improved cardiac function; enhanced repair	[55]
MSC-EVs	hydrogel-based delivery (HA ExoGel); minimally invasive intrapericardial administration (local/retentive delivery)	intrapericardial	Rat pressure-overload HF (TAC)	reduced fibrosis; improved cardiac function; attenuated remodelling	[56]
MSC-EVs	CD47 surface display (MSC CD47 overexpression) to reduce MPS clearance and prolong circulation; miR-21a loading via electroporation (two-step engineered EVs)	IV (tail vein)	Mouse myocardial I/R injury (LAD ligation/reperfusion)	prolonged circulation; increased cardiac accumulation; reduced cardiomyocyte apoptosis; attenuated inflammatory infiltration; improved cardiac function recovery	[57]
GWIT-iCPP-Exo	Source-cell reprogramming via transcription factor overexpression (GLI1/WNT2/ISL1/TBX5); dose strategy (Exo-low vs. Exo-high)	Intratracheal instillation	LPS-induced mouse ALI	dose-dependent attenuation of lung inflammation; improved endothelial function; restored capillary endothelium and epithelial barrier	[58]

**Table 2:** Overview of common EVs isolation and purification methods: applicability, purity, yield, and impact on EV function.

Isolation Method	Principle And Typical Use	Purity (Main Contaminants)	Yield/Cost/Scalability	Impact on EV Integrity and Function	Ref.
Differential ultracentrifugation (dUC)	Sequential low- to high-speed centrifugation to pellet EVs by sedimentation; classical method for cell culture supernatants and some biofluids.	Purity: low–moderate; co-isolation of proteins, lipoproteins, protein/RNA aggregates and mixed EV subtypes.	Yield: medium. Cost: low consumables but requires an ultracentrifuge. Scalability: batch-based, time-consuming for large volumes.	High g-forces and long spins can induce aggregation and partial membrane damage, potentially affecting biodistribution and biological activity.	[73]
Density-gradient ultracentrifugation (DG-UC)	Flotation or sedimentation of EVs through sucrose or iodixanol gradients to separate them from particles of different buoyant densities; often used for plasma/serum when high purity is required.	Purity: generally higher than dUC; improved separation from soluble proteins and part of lipoproteins, but overlapping-density species can remain.	Yield: lower than dUC due to narrower density window and handling losses. Cost: higher (gradient media, tubes, time). Scalability: technically demanding, low–medium throughput.	Gentler than hard pelleting; typically preserves EV markers reasonably well, although long ultracentrifugation still poses some risk to integrity.	[74]
Size-exclusion chromatography (SEC)	Separation by hydrodynamic size on porous matrices (e.g., Sepharose CL-2B); EVs elute in early/void fractions, smaller proteins and many soluble contaminants enter the pores.	Purity: high for soluble proteins; many free proteins and some lipoproteins are efficiently removed, though very small EVs and some lipoprotein particles may overlap.	Yield: moderate, with some dilution and column-binding losses. Cost: columns and buffers moderate; columns often reusable. Scalability: good standardization; throughput can be increased by parallel columns or automation.	Low shear and no extreme g-forces; generally maintains EV morphology, surface markers and cargo, suitable for functional and omics analyses.	[75]
Ultrafiltration/tangential-flow filtration (UF/TFF)	Membrane-based retention of EVs above a defined pore size or MWCO; used to concentrate EVs and often combined with SEC or chromatography for further purification.	Purity: moderate when used alone; removes very large particles and small solutes, but protein aggregates and non-EV nanoparticles can be retained and co-concentrated.	Yield: high and compatible with large volumes. Cost: higher equipment cost but economical for scale. Scalability: excellent; attractive for GMP-scale EV production.	Shear at the membrane can be kept relatively low; when optimized, UF/TFF preserves EV size distribution and protein composition better than dUC.	[76]
Polymer-based precipitation (e.g., PEG; ExoQuick-like)	Hydrophilic polymers (typically PEG) reduce EVs solubility and promote precipitation of EVs together with other macromolecular complexes; convenient, no specialized equipment.	Purity: relatively low; strong co-precipitation of lipoproteins, protein complexes and polymer; often requires additional clean-up for sensitive downstream analyses.	Yield: high particle counts (EVs plus contaminants). Cost: PEG itself inexpensive; commercial kits can be relatively costly per sample. Scalability: simple for many small samples but less convenient for very large volumes.	EVs are usually morphologically intact, but residual polymer and altered protein corona can influence uptake and biological activity; additional purification is recommended for functional studies.	[77]
Immunoaffinity capture	Antibody-coated beads, plates or columns capture EVs expressing specific surface antigens (e.g., CD9, CD63, CD81, EpCAM); used for highly specific enrichment or phenotyping of defined EV subsets.	Purity: very high for the targeted EVs subpopulation; strong depletion of unrelated proteins and EVs, but strong selection bias toward marker-positive vesicles.	Yield: low–moderate and limited to antigen-positive EVs; capacity determined by antibody surface and antigen expression levels. Cost: high due to antibodies and matrices. Scalability: mainly suitable for analytical/diagnostic applications rather than bulk production.	Binding and elution can alter surface epitopes and cargo; harsh elution or on-bead lysis prevents re-use of intact EVs in functional assays, so gentle conditions are required if functional integrity is important.	[78]
Microfluidic-based isolation (emerging)	Lab-on-chip devices that isolate EVs directly from small volumes of biofluids via on-chip immunocapture, size-based filtration or acoustofluidic separation; often integrate capture, washing and detection.	Purity: medium–high, depending on design. Immunocapture chips provide highly enriched subpopulations; acoustofluidic chips deplete cells and platelets and enrich small EVs, though some lipoproteins/protein aggregates may remain.	Yield: moderate and sufficient for diagnostics, limited by chip capacity and channel fouling. Cost: devices may be costly to fabricate, but per-sample reagent cost is low. Scalability: good for parallel processing of many small samples; currently less suited for large therapeutic batches.	Typically low shear and short residence time, preserving EVs morphology and cargo; strong surface interactions and narrow channels can introduce selection bias and potentially alter surface proteins.	[79]
Asymmetric-flow field-flow fractionation (AF4; emerging)	Separation of nanoparticles in a thin channel using a cross-flow field perpendicular to laminar flow; EVs and non-vesicular nanoparticles are resolved by hydrodynamic size and diffusion. Used for high-resolution fractionation and characterization of EVs subpopulations and non-vesicular nanoparticles.	Purity: very high analytical purity and resolution when combined with pre-enrichment (e.g., UC, SEC); efficient separation of EVs from many protein aggregates and lipoproteins and resolution of distinct EVs subsets and non-membranous nanoparticles.	Yield: good particle recovery in collected fractions but mainly analytical rather than preparative at present. Cost: high instrumentation and expertise requirements. Scalability: limited for bulk production; powerful for detailed biophysical and omics studies.	Label-free, low-shear separation without hard pelleting; EVs integrity is generally well preserved. AF4 has revealed size- and compositionally distinct EVs subsets and exomeres with different organ biodistribution and potential biological functions.	[80]

**Table 3:** Overview of Clinical Trials Involving Exosome-Based Therapies in Cardiovascular and Vascular Complications.

Registry ID	Product/EV Source	Indication/Population	Phase and Design	Route and Regimen	Primary Endpoint(s)	Key Exploratory Endpoints	Ref
NCT05774509	EV-enriched secretome from iPSC-derived cardiovascular progenitor cells	HFrEF (non-ischemic DCM)	Phase I, single-group, open-label	IV, 3 infusions; dose-escalation	Serious adverse events	Potency/bioactivity assays; immunologic measures; longer-term follow-up signals	[105]
NCT04327635	EV-containing biological drug (PEP)	PCI ± stent placement	Early-phase, sequential assignment, open-label	Intracoronary, single infusion post-PCI	DLT/MTD (safety/tolerability)	CMR scar size; CMR ejection fraction; alloimmune response	[106]
NCT05669144	MSC-derived exosomes (±mitochondria)	Recent Q-wave MI; CABG candidates; very low LVEF	Phase I/II, randomized, parallel, quadruple-masked	Intracoronary + intramyocardial	EF; allergic reactions	Viability imaging; NYHA class; biomarkers (per protocol)	[107]
NCT02565264	Plasma-derived exosomes	Intractable cutaneous ulcers (vascular-complication context)	Interventional, single-group, open-label	Topical daily × 28 days	Ulcer size metrics	Pain score	[108]

## Data Availability

No new data were created or analyzed in this study. Data sharing is not applicable to this article.

## References

[1] Johansson S., Rosengren A., Young K., Jennings E. (2017). Mortality and morbidity trends after the first year in survivors of acute myocardial infarction: A systematic review. BMC Cardiovasc. Disord..

[2] Butler J., Hammonds K., Talha K.M., Alhamdow A., Bennett M.M., Bomar J.V.A., A Ettlinger J., Traba M.M., Priest E.L., Schmedt N. (2025). Incident heart failure and recurrent coronary events following acute myocardial infarction. Eur. Hear. J..

[3] Rashid M., Abramov D., Naseer M.U., Van Spall H.G.C., Ahmed F.Z., Lawson C., Dafaalla M., Kontopantelis E., O Mohamed M., Petrie M.C. (2025). 15-Year trends, predictors, and outcomes of heart failure hospitalization complicating first acute myocardial infarction in the modern percutaneous coronary intervention era. Eur. Hear. J. Open.

[4] Cung T.-T., Morel O., Cayla G., Rioufol G., Garcia-Dorado D., Angoulvant D., Bonnefoy-Cudraz E., Guérin P., Elbaz M., Delarche N. (2015). Cyclosporine before PCI in Patients with Acute Myocardial Infarction. N. Engl. J. Med..

[5] Hausenloy D.J., Yellon D.M. (2015). Targeting Myocardial Reperfusion Injury—The Search Continues. N. Engl. J. Med..

[6] Hausenloy D.J., Kharbanda R.K., Møller U.K., Ramlall M., Aarøe J., Butler R., Bulluck H., Clayton T., Dana A., Dodd M. (2019). Effect of remote ischaemic conditioning on clinical outcomes in patients with acute myocardial infarction (CONDI-2/ERIC-PPCI): A single-blind randomised controlled trial. Lancet.

[7] Qian G., Zhang Y., Ma Z., Yang R., A X., Tian J., Li P., Zhang H., Ma X., Zhao L. (2025). Long-term prognostic role of persistent microvascular obstruction determined by cardiac magnetic resonance for ST-segment elevation myocardial infarction. Am. Hear. J..

[8] Duan Y., Yin Q., Yang Y., Miao H., Han S., Chi Q., Lv H., Lu Y., Zhou Y. (2025). Integrating angio-IMR and CMR-assessed microvascular obstruction for improved risk stratification of STEMI patients. Sci. Rep..

[9] Ibrahim A.G.-E., Cheng K., Marbán E. (2014). Exosomes as Critical Agents of Cardiac Regeneration Triggered by Cell Therapy. Stem Cell Rep..

[10] Gallet R., Dawkins J., Valle J., Simsolo E., De Couto G., Middleton R., Tseliou E., Luthringer D., Kreke M., Smith R.R. (2017). Exosomes secreted by cardiosphere-derived cells reduce scarring, attenuate adverse remodelling, and improve function in acute and chronic porcine myocardial infarction. Eur. Heart J..

[11] Barile L., Lionetti V., Cervio E., Matteucci M., Gherghiceanu M., Popescu L.M., Torre T., Siclari F., Moccetti T., Vassalli G. (2014). Extracellular vesicles from human cardiac progenitor cells inhibit cardiomyocyte apoptosis and improve cardiac function after myocardial infarction. Cardiovasc. Res..

[12] Mentkowski K.I., Lang J.K. (2019). Exosomes Engineered to Express a Cardiomyocyte Binding Peptide Demonstrate Improved Cardiac Retention in Vivo. Sci. Rep..

[13] Rogers R.G., Ciullo A., Marbán E., Ibrahim A.G. (2020). Extracellular Vesicles as Therapeutic Agents for Cardiac Fibrosis. Front. Physiol..

[14] de Castilla P.E.M., Tong L., Huang C., Sofias A.M., Pastorin G., Chen X., Storm G., Schiffelers R.M., Wang J.-W. (2021). Extracellular vesicles as a drug delivery system: A systematic review of preclinical studies. Adv. Drug Deliv. Rev..

[15] Hu Y., Zhang W., Ali S.R., Takeda K., Vahl T.P., Zhu D., Hong Y., Cheng K. (2024). Extracellular vesicle therapeutics for cardiac repair. J. Mol. Cell. Cardiol..

[16] Kang M., Jordan V., Blenkiron C., Chamley L.W. (2021). Biodistribution of extracellular vesicles following administration into animals: A systematic review. J. Extracell. Vesicles.

[17] Foglio E., Pellegrini L., Russo M.A., Limana F. (2022). HMGB1-Mediated Activation of the Inflammatory-Reparative Response Following Myocardial Infarction. Cells.

[18] Ciortan L., Macarie R.D., Barbu E., Naie M.L., Mihaila A.C., Serbanescu M., Butoi E. (2025). Cross-Talk Between Neutrophils and Macrophages Post-Myocardial Infarction: From Inflammatory Drivers to Therapeutic Targets. Int. J. Mol. Sci..

[19] Yan X., Anzai A., Katsumata Y., Matsuhashi T., Ito K., Endo J., Yamamoto T., Takeshima A., Shinmura K., Shen W. (2013). Temporal dynamics of cardiac immune cell accumulation following acute myocardial infarction. J. Mol. Cell. Cardiol..

[20] Luther K.M., Haar L., McGuinness M., Wang Y., Lynch T.L.L., Phan A., Song Y., Shen Z., Gardner G., Kuffel G. (2018). Exosomal miR-21a-5p mediates cardioprotection by mesenchymal stem cells. J. Mol. Cell. Cardiol..

[21] Cambier L., De Couto G., Ibrahim A., Echavez A.K., Valle J., Liu W., Kreke M., Smith R.R., Marbán L., Marbán E. (2017). Y RNA fragment in extracellular vesicles confers cardioprotection via modulation of IL-10 expression and secretion. EMBO Mol. Med..

[22] Guo H., Li Z., Xiao B., Huang R. (2024). M2 macrophage-derived exosomes promote angiogenesis and improve cardiac function after myocardial infarction. Biol. Direct.

[23] Li D., Zhao Y., Zhang C., Wang F., Zhou Y., Jin S. (2021). Plasma Exosomes at the Late Phase of Remote Ischemic Pre-conditioning Attenuate Myocardial Ischemia-Reperfusion Injury Through Transferring miR-126a-3p. Front. Cardiovasc. Med..

[24] Shafei S., Khanmohammadi M., Ghanbari H., Nooshabadi V.T., Tafti S.H.A., Rabbani S., Kasaiyan M., Basiri M., Tavoosidana G. (2022). Effectiveness of exosome mediated miR-126 and miR-146a delivery on cardiac tissue regeneration. Cell Tissue Res..

[25] Wiklander O.P.B., Nordin J.Z., O’Loughlin A., Gustafsson Y., Corso G., Mäger I., Vader P., Lee Y., Sork H., Seow Y. (2015). Extracellular vesicle in vivo biodistribution is determined by cell source, route of administration and targeting. J. Extracell. Vesicles.

[26] Christianson H.C., Svensson K.J., van Kuppevelt T.H., Li J.-P., Belting M. (2013). Cancer cell exosomes depend on cell-surface heparan sulfate proteoglycans for their internalization and functional activity. Proc. Natl. Acad. Sci. USA.

[27] Hoshino A., Costa-Silva B., Shen T.-L., Rodrigues G., Hashimoto A., Mark M.T., Molina H., Kohsaka S., Di Giannatale A., Ceder S. (2015). Tumour exosome integrins determine organotropic metastasis. Nature.

[28] Cedillo-Servin G., Louro A.F., Gamelas B., Meliciano A., Zijl A., Alves P.M., Malda J., Serra M., Castilho M. (2023). Microfiber-reinforced hydrogels prolong the release of human induced pluripotent stem cell-derived extracellular vesicles to promote endothelial migration. Mater. Sci. Eng. C.

[29] Chen C.W., Wang L.L., Zaman S., Gordon J., Arisi M.F., Venkataraman C.M., Chung J.J., Hung G., Gaffey A.C., Spruce L.A. (2018). Sustained release of endothelial progenitor cell-derived extracellular vesicles from shear-thinning hydrogels improves angiogenesis and promotes function after myocardial infarction. Cardiovasc. Res..

[30] Charles C.J., Li R.R., Yeung T., Mazlan S.M.I., Lai R.C., de Kleijn D.P.V., Lim S.K., Richards A.M. (2020). Systemic Mesenchymal Stem Cell-Derived Exosomes Reduce Myocardial Infarct Size: Characterization With MRI in a Porcine Model. Front. Cardiovasc. Med..

[31] Bian S., Zhang L., Duan L., Wang X., Min Y., Yu H. (2013). Extracellular vesicles derived from human bone marrow mesenchymal stem cells promote angiogenesis in a rat myocardial infarction model. J. Mol. Med..

[32] Sun J., Shen H., Shao L., Teng X., Chen Y., Liu X., Yang Z., Shen Z. (2020). HIF-1α overexpression in mesenchymal stem cell-derived exosomes mediates cardioprotection in myocardial infarction by enhanced angiogenesis. Stem Cell Res. Ther..

[33] Zhu W., Sun L., Zhao P., Liu Y., Zhang J., Zhang Y., Hong Y., Zhu Y., Lu Y., Zhao W. (2021). Macrophage migration inhibitory factor facilitates the therapeutic efficacy of mesenchymal stem cells derived exosomes in acute myocardial infarction through upregulating miR-133a-3p. J. Nanobiotechnol..

[34] Li H., Wang L., Ma T., Liu Z., Gao L. (2023). Exosomes secreted by endothelial cells derived from human induced pluripotent stem cells improve recovery from myocardial infarction in mice. Stem Cell Res. Ther..

[35] Ning H., Chen H., Deng J., Xiao C., Xu M., Shan L., Yang C., Zhang Z. (2021). Exosomes secreted by FNDC5-BMMSCs protect myocardial infarction by anti-inflammation and macrophage polarization via NF-κB signaling pathway and Nrf2/HO-1 axis. Stem Cell Res. Ther..

[36] Ma T., Chen Y., Chen Y., Meng Q., Sun J., Shao L., Yu Y., Huang H., Hu Y., Yang Z. (2018). MicroRNA-132, Delivered by Mesenchymal Stem Cell-Derived Exosomes, Promote Angiogenesis in Myocardial Infarction. Stem Cells Int..

[37] Santoso M.R., Ikeda G., Tada Y., Jung J., Vaskova E., Sierra R.G., Gati C., Goldstone A.B., von Bornstaedt D., Shukla P. (2020). Exosomes From Induced Pluripotent Stem Cell–Derived Cardiomyocytes Promote Autophagy for Myocardial Repair. J. Am. Hear. Assoc..

[38] Minghua W., Zhijian G., Chahua H., Qiang L., Minxuan X., Luqiao W., Weifang Z., Peng L., Biming Z., Lingling Y. (2018). Plasma exosomes induced by remote ischaemic preconditioning attenuate myocardial ischaemia/reperfusion injury by transferring miR-24. Cell Death Dis..

[39] Luo Q., Guo D., Liu G., Chen G., Hang M., Jin M. (2017). Exosomes from MiR-126-Overexpressing Adscs Are Therapeutic in Relieving Acute Myocardial Ischaemic Injury. Cell. Physiol. Biochem..

[40] Cheng H., Chang S., Xu R., Chen L., Song X., Wu J., Qian J., Zou Y., Ma J. (2020). Hypoxia-challenged MSC-derived exosomes deliver miR-210 to attenuate post-infarction cardiac apoptosis. Stem Cell Res. Ther..

[41] Zhu L.-P., Tian T., Wang J.-Y., He J.-N., Chen T., Pan M., Xu L., Zhang H.-X., Qiu X.-T., Li C.-C. (2018). Hypoxia-elicited mesenchymal stem cell-derived exosomes facilitates cardiac repair through miR-125b-mediated prevention of cell death in myocardial infarction. Theranostics.

[42] Pham T.P., Boon R.A. (2019). Exosomes and non-coding RNA, the healers of the heart?. Cardiovasc. Res..

[43] Ning Y., Huang P., Chen G., Xiong Y., Gong Z., Wu C., Xu J., Jiang W., Li X., Tang R. (2023). Atorvastatin-pretreated mesenchymal stem cell-derived extracellular vesicles promote cardiac repair after myocardial infarction via shifting macrophage polarization by targeting microRNA-139-3p/Stat1 pathway. BMC Med..

[44] Xiong Y., Tang R., Xu J., Jiang W., Gong Z., Zhang L., Ning Y., Huang P., Xu J., Chen G. (2022). Tongxinluo-pretreated mesenchymal stem cells facilitate cardiac repair via exosomal transfer of miR-146a-5p targeting IRAK1/NF-κB p65 pathway. Stem Cell Res. Ther..

[45] Pu Y., Li C., Qi X., Xu R., Dong L., Jiang Y., Gong Q., Wang D., Cheng R., Zhang C. (2023). Extracellular Vesicles from NMN Preconditioned Mesenchymal Stem Cells Ameliorated Myocardial Infarction via miR-210-3p Promoted Angiogenesis. Stem Cell Rev. Rep..

[46] Song Y., Zhang C., Zhang J., Jiao Z., Dong N., Wang G., Wang Z., Wang L. (2019). Localized injection of miRNA-21-enriched extracellular vesicles effectively restores cardiac function after myocardial infarction. Theranostics.

[47] Zhu W., Wang Q., Zhang J., Sun L., Hong X., Du W., Duan R., Jiang J., Ji Y., Wang H. (2023). Exosomes derived from mir-214-3p overexpressing mesenchymal stem cells promote myocardial repair. Biomater. Res..

[48] Gong Z.-T., Xiong Y.-Y., Ning Y., Tang R.-J., Xu J.-Y., Jiang W.-Y., Li X.-S., Zhang L.-L., Chen C., Pan Q. (2024). Nicorandil-Pretreated Mesenchymal Stem Cell-Derived Exosomes Facilitate Cardiac Repair After Myocardial Infarction via Promoting Macrophage M2 Polarization by Targeting miR-125a-5p/TRAF6/IRF5 Signaling Pathway. Int. J. Nanomed..

[49] Ciullo A., Biemmi V., Milano G., Bolis S., Cervio E., Fertig E.T., Gherghiceanu M., Moccetti T., Camici G.G., Vassalli G. (2019). Exosomal Expression of CXCR4 Targets Cardioprotective Vesicles to Myocardial Infarction and Improves Outcome after Systemic Administration. Int. J. Mol. Sci..

[50] Xiao Z., Li S., Wu X., Chen X., Yan D., He J. (2024). GATA-4 overexpressing BMSC-derived exosomes suppress H/R-induced cardiomyocyte ferroptosis. iScience.

[51] He J.-G., Li H.-R., Han J.-X., Li B.-B., Yan D., Li H.-Y., Wang P., Luo Y. (2018). GATA-4-expressing mouse bone marrow mesenchymal stem cells improve cardiac function after myocardial infarction via secreted exosomes. Sci. Rep..

[52] Kang J.-Y., Kim H., Mun D., Yun N., Joung B. (2021). Co-delivery of curcumin and miRNA-144-3p using heart-targeted extracellular vesicles enhances the therapeutic efficacy for myocardial infarction. J. Control. Release.

[53] Zhang J., Zhang B., Zhang L., Xu X., Cheng Q., Wang Y., Li Y., Jiang R., Duan S., Zhang L. (2024). Engineered nanovesicles mediated cardiomyocyte survival and neovascularization for the therapy of myocardial infarction. Colloids Surf. B Biointerfaces.

[54] Wang X., Chen Y., Zhao Z., Meng Q., Yu Y., Sun J., Yang Z., Chen Y., Li J., Ma T. (2018). Engineered Exosomes With Ischemic Myocardium-Targeting Peptide for Targeted Therapy in Myocardial Infarction. J. Am. Hear. Assoc..

[55] Wang J., Li J., Su G., Zhang Y., Wang Z., Jia Y., Yu Q., Shen Z., Zhang Y., Yu Y. (2024). Neutrophil-derived apoptotic body membranes-fused exosomes targeting treatment for myocardial infarction. Regen. Biomater..

[56] Cheng G., Zhu D., Huang K., Caranasos T.G. (2022). Minimally invasive delivery of a hydrogel-based exosome patch to prevent heart failure. J. Mol. Cell. Cardiol..

[57] Wei Z., Chen Z., Zhao Y., Fan F., Xiong W., Song S., Yin Y., Hu J., Yang K., Yang L. (2021). Mononuclear phagocyte system blockade using extracellular vesicles modified with CD47 on membrane surface for myocardial infarction reperfusion injury treatment. Biomaterials.

[58] Xia L.-X., Xiao Y.-Y., Jiang W.-J., Yang X.-Y., Tao H., Mandukhail S.R., Qin J.-F., Pan Q.-R., Zhu Y.-G., Zhao L.-X. (2024). Exosomes derived from induced cardiopulmonary progenitor cells alleviate acute lung injury in mice. Acta Pharmacol. Sin..

[59] Gao L., Wang L., Wei Y., Krishnamurthy P., Walcott G.P., Menasché P., Zhang J. (2020). Exosomes secreted by hiPSC-derived cardiac cells improve recovery from myocardial infarction in swine. Sci. Transl. Med..

[60] Kim S.-C., Stice J.P., Chen L., Jung J.S., Gupta S., Wang Y., Baumgarten G., Trial J., Knowlton A.A. (2009). Extracellular Heat Shock Protein 60, Cardiac Myocytes, and Apoptosis. Circ. Res..

[61] Malik Z.A., Kott K.S., Poe A.J., Kuo T., Chen L., Ferrara K.W., Knowlton A.A. (2013). Cardiac myocyte exosomes: Stability, HSP60, and proteomics. Am. J. Physiol. Circ. Physiol..

[62] Qiao L., Hu S., Liu S., Zhang H., Ma H., Huang K., Li Z., Su T., Vandergriff A., Tang J. (2019). microRNA-21-5p dysregulation in exosomes derived from heart failure patients impairs regenerative potential. J. Clin. Investig..

[63] Ji Z., Wang C. (2024). Mesenchymal stem cell-derived exosomal mir-21-5p inhibits YAP1 expression and improves outcomes in myocardial infarction. BMC Cardiovasc. Disord..

[64] Vandergriff A., Huang K., Shen D., Hu S., Hensley M.T., Caranasos T.G., Qian L., Cheng K. (2018). Targeting regenerative exosomes to myocardial infarction using cardiac homing peptide. Theranostics.

[65] He L., He T., Xing J., Zhou Q., Fan L., Liu C., Chen Y., Wu D., Tian Z., Liu B. (2020). Bone marrow mesenchymal stem cell-derived exosomes protect cartilage damage and relieve knee osteoarthritis pain in a rat model of osteoarthritis. Stem Cell Res. Ther..

[66] Wong K.L., Zhang S., Wang M., Ren X., Afizah H., Lai R.C., Lim S.K., Lee E.H., Hui J.H.P., Toh W.S. (2020). Intra-Articular Injections of Mesenchymal Stem Cell Exosomes and Hyaluronic Acid Improve Structural and Mechanical Properties of Repaired Cartilage in a Rabbit Model. Arthrosc. J. Arthrosc. Relat. Surg..

[67] Gharehchelou B., Mehrarya M., Sefidbakht Y., Uskoković V., Suri F., Arjmand S., Maghami F., Siadat S.O.R., Karima S., Vosough M. (2025). Mesenchymal stem cell-derived exosome and liposome hybrids as transfection nanocarriers of Cas9-GFP plasmid to HEK293T cells. PLoS ONE.

[68] Villa C., Secchi V., Macchi M., Tripodi L., Trombetta E., Zambroni D., Padelli F., Mauri M., Molinaro M., Oddone R. (2024). Magnetic-field-driven targeting of exosomes modulates immune and metabolic changes in dystrophic muscle. Nat. Nanotechnol..

[69] Hu S., Wang X., Li Z., Zhu D., Cores J., Wang Z., Li J., Mei X., Cheng X., Su T. (2021). Platelet membrane and stem cell exosome hybrids enhance cellular uptake and targeting to heart injury. Nano Today.

[70] Haraszti R.A., Miller R., Stoppato M., Sere Y.Y., Coles A., Didiot M.-C., Wollacott R., Sapp E., Dubuke M.L., Li X. (2018). Exosomes Produced from 3D Cultures of MSCs by Tangential Flow Filtration Show Higher Yield and Improved Activity. Mol. Ther..

[71] Ulpiano C., Salvador W., Franchi-Mendes T., Huang M.-C., Lin Y.-H., Lin H.-T., Rodrigues C.A.V., Fernandes-Platzgummer A., Cabral J.M.S., Monteiro G.A. (2025). Continuous collection of human mesenchymal-stromal-cell-derived extracellular vesicles from a stirred tank reactor operated under xenogeneic-free conditions for therapeutic applications. Stem Cell Res. Ther..

[72] Welsh J.A., Goberdhan D.C., O’Driscoll L., Théry C., Witwer K.W. (2024). MISEV2023: An updated guide to EV research and applications. J. Extracell. Vesicles.

[73] Théry C., Witwer K.W., Aikawa E., Alcaraz M.J., Anderson J.D., Andriantsitohaina R., Antoniou A., Arab T., Archer F., Atkin-Smith G.K. (2018). Minimal information for studies of extracellular vesicles 2018 (MISEV2018): A position statement of the International Society for Extracellular Vesicles and update of the MISEV2014 guidelines. J. Extracell. Vesicles.

[74] Onodi Z., Pelyhe C., Terezia Nagy C., Brenner G.B., Almasi L., Kittel A., Mancek-Keber M., Ferdinandy P., Buzas E.I., Giricz Z. (2018). Isolation of High-Purity Extracellular Vesicles by the Combination of Iodixanol Density Gradient Ultracentrifugation and Bind-Elute Chromatography From Blood Plasma. Front. Physiol..

[75] Böing A.N., van der Pol E., Grootemaat A.E., Coumans F.A.W., Sturk A., Nieuwland R. (2014). Single-step isolation of extracellular vesicles by size-exclusion chromatography. J. Extracell. Vesicles.

[76] Busatto S., Vilanilam G., Ticer T., Lin W.-L., Dickson D.W., Shapiro S., Bergese P., Wolfram J. (2018). Tangential Flow Filtration for Highly Efficient Concentration of Extracellular Vesicles from Large Volumes of Fluid. Cells.

[77] Gámez-Valero A., Monguió-Tortajada M., Carreras-Planella L., La Franquesa M., Beyer K., Borràs F.E. (2016). Size-Exclusion Chromatography-based isolation minimally alters Extracellular Vesicles’ characteristics compared to precipitating agents. Sci. Rep..

[78] Ströhle G., Gan J., Li H. (2022). Affinity-based isolation of extracellular vesicles and the effects on downstream molecular analysis. Anal. Bioanal. Chem..

[79] Meggiolaro A., Moccia V., Brun P., Pierno M., Mistura G., Zappulli V., Ferraro D. (2022). Microfluidic Strategies for Extracellular Vesicle Isolation: Towards Clinical Applications. Biosensors.

[80] Zhang H., Freitas D., Kim H.S., Fabijanic K., Li Z., Chen H., Mark M.T., Molina H., Martin A.B., Bojmar L. (2018). Identification of distinct nanoparticles and subsets of extracellular vesicles by asymmetric flow field-flow fractionation. Nat. Cell Biol..

[81] Yan L., Wu X. (2019). Exosomes produced from 3D cultures of umbilical cord mesenchymal stem cells in a hollow-fiber bioreactor show improved osteochondral regeneration activity. Cell Biol. Toxicol..

[82] Takov K., Yellon D.M., Davidson S.M. (2019). Comparison of small extracellular vesicles isolated from plasma by ultracentrifugation or size-exclusion chromatography: Yield, purity and functional potential. J. Extracell. Vesicles.

[83] Nordin J.Z., Lee Y., Vader P., Mäger I., Johansson H.J., Heusermann W., Wiklander O.P.B., Hällbrink M., Seow Y., Bultema J.J. (2015). Ultrafiltration with size-exclusion liquid chromatography for high yield isolation of extracellular vesicles preserving intact biophysical and functional properties. Nanomed. Nanotechnol. Biol. Med..

[84] Ter-Ovanesyan D., Gilboa T., Budnik B., Nikitina A., Whiteman S., Lazarovits R., Trieu W., Kalish D., Church G.M., Walt D.R. (2023). Improved isolation of extracellular vesicles by removal of both free proteins and lipoproteins. eLife.

[85] Xu R., Greening D.W., Zhu H.-J., Takahashi N., Simpson R.J. (2016). Extracellular vesicle isolation and characterization: Toward clinical application. J. Clin. Investig..

[86] Wu M., Chen C., Wang Z., Bachman H., Ouyang Y., Huang P.-H., Sadovsky Y., Huang T.J. (2019). Separating extracellular vesicles and lipoproteins *via* acoustofluidics. Lab A Chip.

[87] Li M., Soder R., Abhyankar S., Home T., Pathak H., Shen X., Godwin A.K., Abdelhakim H. (2025). Large-scale manufacturing of immunosuppressive extracellular vesicles for human clinical trials. Cytotherapy.

[88] Gobin J., Muradia G., Mehic J., Westwood C., Couvrette L., Stalker A., Bigelow S., Luebbert C.C., Bissonnette F.S.-D., Johnston M.J.W. (2021). Hollow-fiber bioreactor production of extracellular vesicles from human bone marrow mesenchymal stromal cells yields nanovesicles that mirrors the immuno-modulatory antigenic signature of the producer cell. Stem Cell Res. Ther..

[89] Powsner E.H., Kronstadt S.M., Nikolov K., Aranda A., Jay S.M. (2025). Mesenchymal stem cell extracellular vesicle vascularization bioactivity and production yield are responsive to cell culture substrate stiffness. Bioeng. Transl. Med..

[90] Garcia S.G., Clos-Sansalvador M., Sanroque-Muñoz M., Pan L., Franquesa M. (2024). Functional and potency assays for mesenchymal stromal cell–extracellular vesicles in kidney disease. Curr. Opin. Physiol..

[91] Kobayashi H., Shiba T., Yoshida T., Bolidong D., Kato K., Sato Y., Mochizuki M., Seto T., Kawashiri S., Hanayama R. (2024). Precise analysis of single small extracellular vesicles using flow cytometry. Sci. Rep..

[92] Liu H., Tian Y., Xue C., Niu Q., Chen C., Yan X. (2022). Analysis of extracellular vesicle DNA at the single-vesicle level by nano-flow cytometry. J. Extracell. Vesicles.

[93] Mizenko R.R., Brostoff T., Rojalin T., Koster H.J., Swindell H.S., Leiserowitz G.S., Wang A., Carney R.P. (2021). Tetraspanins are unevenly distributed across single extracellular vesicles and bias sensitivity to multiplexed cancer biomarkers. J. Nanobiotechnol..

[94] Ashique S., Anand K. (2023). Radiolabelled Extracellular Vesicles as Imaging Modalities for Precise Targeted Drug Delivery. Pharmaceutics.

[95] Ibanez B., Aletras A.H., Arai A.E., Arheden H., Bax J., Berry C., Bucciarelli-Ducci C., Croisille P., Dall’Armellina E., Dharmakumar R. (2019). Cardiac MRI Endpoints in Myocardial Infarction Experimental and Clinical Trials: JACC Scientific Expert Panel. J. Am. Coll. Cardiol..

[96] Patel S., Schmidt K.F., Farhoud M., Zi T., Jang S.C., Dooley K., Kentala D., Dobson H., Economides K., Williams D.E. (2022). In vivo tracking of [89Zr]Zr-labeled engineered extracellular vesicles by PET reveals organ-specific biodistribution based upon the route of administration. Nucl. Med. Biol..

[97] Hikita T., Miyata M., Watanabe R., Oneyama C. (2020). In vivo imaging of long-term accumulation of cancer-derived exosomes using a BRET-based reporter. Sci. Rep..

[98] Leal A.C., Mizurini D.M., Gomes T., Rochael N.C., Saraiva E.M., Dias M.S., Werneck C.C., Sielski M.S., Vicente C.P., Monteiro R.Q. (2017). Tumor-Derived Exosomes Induce the Formation of Neutrophil Extracellular Traps: Implications For The Establishment of Cancer-Associated Thrombosis. Sci. Rep..

[99] Almeida V.H., Rondon A.M.R., Gomes T., Monteiro R.Q. (2019). Novel Aspects of Extracellular Vesicles as Mediators of Cancer-Associated Thrombosis. Cells.

[100] Sun L., Ji Y., Chi B., Xiao T., Li C., Yan X., Xiong X., Mao L., Cai D., Zou A. (2023). A 3D culture system improves the yield of MSCs-derived extracellular vesicles and enhances their therapeutic efficacy for heart repair. Biomed. Pharmacother..

[101] Bauer F.N., Tertel T., Stambouli O., Wang C., Dittrich R., Staubach S., Börger V., Hermann D.M., Brandau S., Giebel B. (2022). CD73 activity of mesenchymal stromal cell-derived extracellular vesicle preparations is detergent-resistant and does not correlate with immunomodulatory capabilities. Cytotherapy.

[102] Mizenko R.R., Feaver M., Bozkurt B.T., Lowe N., Nguyen B., Huang K., Wang A., Carney R.P. (2024). A critical systematic review of extracellular vesicle clinical trials. J. Extracell. Vesicles.

[103] Giebel B., Lim S.K. (2025). Overcoming challenges in MSC-sEV therapeutics: Insights and advances after a decade of research. Cytotherapy.

[104] Manno M., Bongiovanni A., Margolis L., Bergese P., Arosio P. (2024). The physico-chemical landscape of extracellular vesicles. Nat. Rev. Bioeng..

[105] Assistance Publique-Hôpitaux de Paris Treatment of Non-Ischemic Dilated Cardiomyopathies by Intravenous Infusions of the Extracellular Vesicle-Enriched Secretome of Cardiovascular Progenitor Cells; Clinical Trial Registration NCT05774509; Clinicaltrials.gov, 2023. NCT05774509.

[106] McLeod C.J. Safety Evaluation of Intracoronary Infusion of Extracellular Vesicles in Patients Following Coronary Stent Implantation (EV-CSI); Clinical Trial Registration NCT04327635; Clinicaltrials.gov, 2025. NCT04327635.

[107] tafti A. Co-Transplantation of Mesenchymal Stem Cell Derived Exosomes and Autologous Mitochondria for Patients Candidate for CABG Surgery with EF < 25% (Clinical Trial Phase); Clinical Trial Registration NCT05669144; Clinicaltrials.gov, 2022. NCT05669144.

[108] Kumamoto University Effect of Plasma Derived Exosomes on Intractable Cutaneous Wound Healing: Prospective Trial; Clinical Trial Registration NCT02565264; Clinicaltrials.gov, 2020. NCT02565264.

